# Two murine models of sepsis: immunopathological differences between the sexes—possible role of TGFβ1 in female resistance to endotoxemia

**DOI:** 10.1186/s40659-023-00469-8

**Published:** 2023-10-24

**Authors:** Rafael Bojalil, Armando Ruíz-Hernández, Arturo Villanueva-Arias, Luis Manuel Amezcua-Guerra, Sergio Cásarez-Alvarado, Ana María Hernández-Dueñas, Verónica Rodríguez-Galicia, Lenin Pavón, Brenda Marquina, Enrique Becerril-Villanueva, Rogelio Hernández-Pando, Ricardo Márquez-Velasco

**Affiliations:** 1 Departamento de Inmunología, Instituto Nacional de Cardiología Ignacio Chávez, Mexico City, Mexico; 2 Departamento de Atención a la Salud, Universidad Autónoma Metropolitana-Xochimilco, Mexico City, Mexico; 3 Departamento de Farmacología, Facultad de Medicina, Universidad Autónoma de Baja California, Mexicali, Mexico; 4 Departamento de Microbiología, Instituto Nacional de Cardiología Ignacio Chávez, Mexico City, Mexico; 5 Laboratorio de Psicoinmunología, Instituto Nacional de Psiquiatría Ramón de la Fuente, Mexico City, Mexico; 6 Departamento de Patología Experimental, Instituto Nacional de Ciencias Médicas y Nutrición Salvador Zubirán, Mexico City, Mexico; 7 Departamento de Producción Agrícola y Animal, Universidad Autónoma Metropolitana-Xochimilco, Mexico City, Mexico

## Abstract

Endotoxic shock (ExSh) and cecal ligature and puncture (CLP) are models that induce sepsis. In this work, we investigated early immunologic and histopathologic changes induced by ExSh or CLP models in female and male mice. Remarkable results showed that females supported twice the LD100 of LPS for males, CLP survival and CFU counts were similar between genders, high circulating LPS levels in ExSh mice and low levels of IgM anti-LPS in males. In the serum of ExSh males, TNF and IL-6 increased in the first 6 h, in CLP males at 12 h. In the liver of ExSh mice, TNF increased at 1.5 and 12 h, IL-1 at 6 h. TGFβ1 increased in females throughout the study and at 12 h in males. In CLP mice, IL-6 decreased at 12 h, TGFβ1 increased at 6–12 h in males and at 12 h in females. In the lungs of ExSh males, IL-1β increased at 1.5-6 h and TGFβ1 at 12 h; in females, TNF decrease at 6 h and TGFβ1 increased from 6 h; in CLP females, TNF and IL-1β decreased at 12 h and 1.5 h, respectively, and TGFβ1 increased from 6 h; in males, TGFβ1 increased at 12 h. In the livers of ExSh mice, signs of inflammation were more common in males; in the CLP groups, inflammation was similar but less pronounced. ExSh females had leucocytes with TGFβ1. The lungs of ExSh males showed patches of hyaline membranes and some areas of inflammatory cells, similar but fewer and smaller lesions were seen in male mice with CLP. In ExSh females, injuries were less extent than in males, similar pulmonary lesions were seen in female mice with CLP. ExSh males had lower levels of TGFβ1 than females, and even lower levels were seen in CLP males. We conclude that the ExSh was the most lethal model in males, associated with high levels of free LPS, low IgM anti-LPS, exacerbated inflammation and target organ injury, while females showed early TGFβ1 production in the lungs and less tissue damage. We didn't see any differences between CLP mice.

## Background

Sepsis is defined as life-threatening organ dysfunction caused by a deregulated host response to infection, while septic shock is a subset of sepsis in which the underlying circulatory and cellular/metabolic abnormalities are profound enough to significantly increase mortality [1]. The incidence of sepsis in the United States is approximately one million cases per year, resulting in 200,000 deaths [2]. A multinational study estimated 48.9 million cases of sepsis and 11 million sepsis-related deaths in 2017 [3]. The calculated annual cost of sepsis without organ failure is approximately $22 billion, $10.2 billion for severe sepsis and $19.8 billion for septic shock [4]. Regarding the pathogenesis of sepsis, it begins with microbial entry into the circulation, bacterial antigens such as lipopolysaccharide (LPS) induce overproduction of TNF, IL-1 and IL-6 by macrophages/monocytes, dendritic cells, polymorphonuclear cells, endothelial cells, hepatocytes, etc. [5, 6]. The combination of inflammatory mediators triggers hypotension, decreased peripheral vascular resistance, disseminated intravascular coagulation, tissue hypoxia, overproduction of reactive oxygen species, etc., mechanisms that collectively lead to tissue injury, multiple organ failure, and death [5]. In the laboratory, many immunopathological mechanisms of sepsis have been described in mouse models such as endotoxic shock (ExSh) or cecal ligature and puncture (CLP). The first model is induced by intravenous or intraperitoneal injection of LPS, causing a transient but exaggerated production of proinflammatory cytokines [7], and the second model is induced by a continuous flow of intestinal contents into the peritoneal cavity as a result of intestinal perforation, resulting in a polymicrobial insult and detectable bacteremia, an increase in inflammatory mediators, and the hyper- then hypodynamic transition [8]. Gender differences in susceptibility to sepsis models have been suggested; in a review by Angele MK et al. [9], the authors found that female mice in proestrus had a significantly increased survival rate in the CLP model compared to male mice, while in the ExSh model, female mice were clearly more resistant than male mice. In this context, evidence suggests that the balance of sex hormones plays a central role in infections, because progesterone favors the regulation of the immunological response and protection against infectious diseases [10, 11] by reducing inflammatory cytokines, IL-6 and TNF, and restoring the antioxidant defense system, whereas testosterone contributes to the reduction of the immune response, facilitating the development of infections, as has been demonstrated in orchiectomized animals that were more resistant to this type of infection [12].

Despite this evidence, the contribution of sex hormones to infection susceptibility remains controversial, suggesting that other mechanisms contribute to resistance or susceptibility and outcome in sepsis. In this regard, the ex vivo cells obtained from female mice and cultured in sex hormone-free medium developed a different immune response than cells from male mice, supporting that other immunological regulatory elements contribute to the differences in immune cellular response [13]. Under the assumption that early injury mechanisms in sepsis influence the development of the disease, in this study we evaluated early differences in local and systemic immune mechanisms in both sexes and in ExSh or CLP models, and further evaluated histopathologic differences in target organs.

## Results

**Fig. 1:**
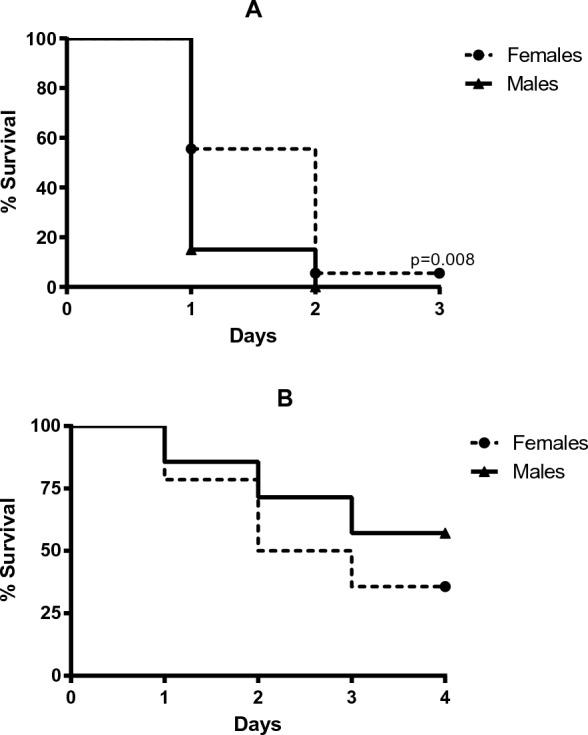
Survival. Survival in **A** endotoxic shock model and **B** CLP model was evaluated one week after challenge, the N in each group was 10 animals. Dotted lines represent female mice, while solid lines represent male mice. Comparisons of survival curves were performed using the Mantel-Haenzel log-rank test. Differences were considered when p < 0.05

Survival. High lethality was documented in ExSh mice (Fig. 1, panel A). In this model, the mortality rate of male mice was 100% two days after LPS challenge, while female mice showed a significant survival rate of about 5% on day 3 (p = 0.008). In contrast, the mortality of both sexes of mice (Fig. 1, panel B) after CLP with the 21G needle was close to 50%.

Colony forming units counts (CFU), LPS levels and anti-LPS IgM. As expected, bacterial blood cultures from the ExSh groups were negative (data not shown). Although there was a higher number of CFU in CLP male mice, 39 (0–1100) (n = 6), compared to female mice, 0 (0–80) (n = 7) (Table 1), the difference was not significant. LPS levels in ExSh mice were around ten times higher (p < 0.5) in both males [1.934 (1.19–1.94 EU/mL)] and females [1.932 (1.83–2.03 EU/mL)] than in their respective control groups [0.264 (0.19–0.33) and 0.175 (0.17–0.22)]. LPS levels in CLP groups were not different from control mice (Table 1). In normal mice, the concentration of IgM anti-LPS antibodies was 12- and sixfold higher in males and females, respectively, than in ExSh mice: 0.49 (0.27–0–74) vs. 0.039 (0.03–0.06) (p < 0.005) in males and 0.27 (0.25–0.50) vs. 0.04 (0.04–0.08) (ns) in females. Normal mice had 2- to threefold higher concentrations of IgM anti-LPS antibodies compared to CLP mice: 0.17 (0.10–0.21) in males and 0.11 (0.08–0.12) in females. IgM levels in male CLP mice were significantly higher than those in male ExSh mice (p < 0.05) (Table 1).

**Table 1:** Determination of CFU, LPS and IgM in in Endotoxemia or CLP models

Analyte	Normal Median (Min–Max)		ExSh Median (Min–Max)		CLP Median (Min–Max)	
	Male	Female	Male	Female	Male	Female
CFU/mL	–	–	–	–	39 (0–1100)	0 (0–80)
LPS (EU/mL)	0.264 (0.19–0.33)	0.175 ( 0.17–0.22)	1.934 (1.19–1.94)*	1.932 (1.83–2.03) *	0.25 (0.13–0.43)	0.379 (0.02–0.05)
IgM (OD)	0.49 (0.27–0.74)	0.27 (0.25–0.50)	0.039 (0.03–0.06)**	0.04 (0.04–0.08)	0.17 (0.10–0.21)+	0.11 (0.08–0.12)

CFU (Colony-forming Unit), mL (mililiter), LPS (Lipopolysaccharide), EU (Endotoxin Unit), IgM (Immunoglobulin M), OD (Optical Density). Statistical differences were considered when *p < 0.05, **p < 0.005 vs Normal group or + p < 0.05 vs ExSh Male. N = 6–7 mice per group to CFU. N = 4–5 mice per group to LPS. N = 4–5 mice per group to IgM

Cytokines in sera. We didn't find any TNF, IL-1, IL-6, or IL-10 in the normal groups. However, TGFβ1 levels were higher than most measurements in the experimental groups, although they were within an extremely wide range: 15–37,454 pg/mL in female and 15–53,470 pg/mL in male mice (Table 2). LPS challenge induced detectable levels of all cytokines measured, however statistical significance was seen only for TNF at 1.5 h and for IL-6 at 6 h post challenge in male mice. TNF levels peaked at 1.5 h and declined rapidly thereafter in both sexes (Table 2). We found the highest levels of IL-1 at 12 h post challenge in male mice; detection was intermittent with an early increase at 1.5 h and no detection at 6 h. In response to LPS, we detected IL-6 beginning at 1.5 h and peaking at 6 h in both sexes but significant only in males, and discrete levels at 12 h. We found that IL-10 and TGFβ peaked at 1.5 h in male mice, earlier than in female mice, which peaked at 12 h (Table 2). There was no evidence of TNF or IL-1 after CLP. IL-6 peaked at 6 h after surgery in female mice and a significant increase at 12 h in male mice. As seen after LPS challenge, IL-10 peaked at 1.5 h after surgery in male mice and at 12 h in female mice (Table 2). After CLP, TGFβ levels peaked at 12 h in male mice and remained stable over time in female mice (Table 2).

**Table 2:** Levels of TNF, IL-1, IL-6, IL-10 and TGFβ1 in Serum

Group	Cytokine (pg/mL)	1.5h Median (Min–Max)		6h Median (Min–Max)		12h Median (Min–Max)	
		Male	Female	Male	Female	Male	Female
Normal	TNF IL-1 IL-6 IL-10 TGFβ	ND ND ND ND 31294 (15–53470)	ND ND ND ND 11934 (15–37454)	–– – – – –	– – – – –	– – – – –	– – – – –
ExSh	TNF IL-1 IL-6 IL-10 TGFβ	6207 (2670–7370)* 160 (15–2320) 11020 (6110–22100) 2260 (440–3000) 20283 (2700–32213)	1006 (15–2108) 16 (15-880) 15080 (480–20380) 16 (15–1400) 2700 (2639–30533)	266 (15–363) ND 18980 (15800–27680)* 670 (15–8490) 5664 (2700–10873)	183 (15–349) ND 26180 (15–26180) 670 (15–1980) 13729 (10537–17090)	ND 1270 (180-3360) 4280 (15–14720) 16 (15–930) 6830 (846–39629)	16 ( 15–970) 16 (15-5380) 12620 (15–30140) 950 (15–3400) 30150 (7300–42558)
CLP	TNF IL-1 IL-6 IL-10 TGFβ	ND ND 16 (15–640) 2265 (510–6250) 2700 (2700–6504)	ND ND 960 (15–28720) ND 2700 (622–2700)	ND ND 16 (15–660) ND 2750 (2700–2780)	ND ND 1520 (15-4820) 16 (16–220) 2700 (2700-6336)	ND ND 1700 (660-9000)* 16 (15–3090) 11406 (2606-13166)	ND ND 16 (15-480) ND 2958 (1022–86030)

*TNF* Tumoral Necrosis Factor, *IL-1* Interleukin 1, *IL-6* Interleukin 6, *IL-10* Interleukin 10, *TGFβ1* Transforming Growth Factor beta

Statistical differences were considered when *p < 0.05 vs Normal group. N = 4–5 mice per group

Cytokines in liver. In the liver, all cytokines were detected in N groups without sex differences (Table 3). Compared with normal mice, female mice after LPS challenge showed a significant increase in TNF at 1.5 h and 12 h, IL-1 at 6 h and 12 h, and TGFβ1 at 1.5 h, 6 h, and 12 h, in an increasing pattern, in all cases, the highest levels were found at 12 h. Male mice showed a significant increase in TNF at 1.5 h and 12 h; IL-1 at 6 h; TGFβ1 also had an increasing pattern reaching significance at 12 h (Table 3).

**Table 3:** Levels of TNF, IL-1, IL-6, IL-10 and TGFβ1 in Liver

Group	Cytokine (pg/mL)	1.5h Median (Min–Max)		6h Median (Min–Max)		12h Median (Min–Max)	
		Male	Female	Male	Female	Male	Female
Normal	TNF IL-1 IL-6 IL-10 TGFβ	36 (17–64) 146 (79–340) 630 (224–1781) 689 (176–1879) 240 (88–639)	24 (15–81) 153 (85–449) 505 (15–2834) 320 (32–867) 198 (118–565)	– – – – –	– – – – –	– – – – –	– – – – –
ExSh	TNF IL-1 IL-6 IL-10 TGFβ	221 (177–500)* 797 (199–1000) 85 (51–147) 478 (243–660) 335 (269–734)	185 (98–443)* 554 (37–1101) 88 (59–178) 247 (225–697) 2299 (1098–3351)*	164 (144–193) 1070 (519–1160)** 100 (79–105) 570 (384–633) 4494 (1227–4914)	167 (121–180) 1021 (433–1088)* 62 (38–98) 356 (339–398) 7579 (3319–13409)*	244 (222–325)*** 341 (173–438) 138 (112–184) 933 (883–1482) 26056 (21223–27090)*	468 (188–730)** 2065 (163–2495)* 143 (15–538) 615 (246–866) 15788 (8742–19256)*
CLP	TNF IL-1 IL-6 IL-10 TGFβ	ND** 219 (134–518) 39 (34–71) 338 (253–501) 1393 (417–2243)	ND** 172 (131–270) 66 (32–90) 506 (142–676) 223 (187–1455)	ND** 190 (145–310) 59 (48–74) 53 (285–945) 4079 (2177–14098)*	ND** 327 (156–530) 87 (22–132) 521 (284–640) 358 (299–538)	ND** 53 (38–95) 24 (11–39)* 293 (115–340) 30533(2658–102584)*	ND** 53 (43–129) 17 (15–22)* 180 (91–348) 40609 (11412–55693)*

*TNF* Tumoral Necrosis Factor, *IL-1* Interleukin 1, *IL-6* Interleukin 6, *IL-10* Interleukin 10, *TGFβ1* Transforming Growth Factor beta

Statistical differences were considered when *p < 0.05, **p < 0.005, ***p<0.0005 vs Normal group. N = 15 mice per N group, N = 4–5 mice per experimental group

After CLP, no TNF was detected in experimental mice (p < 0.005 vs. N mice). Compared with normal mice, female mice also showed a significant decrease in IL-6 at 12 h; and a constant increase in TGFβ1, which reached significance at 12 h. Male mice also showed a significant decrease in IL-6 at 12 h; and a constant increase in TGFβ1, which reached significance at 6 h and 12 h (Table 3).

Lung Cytokines. In the lung, all cytokines measured were detected in the N groups, with no differences between the sexes (Table 4).

**Table 4:** Levels of TNF, IL-1, IL-6, IL-10 and TGFβ1 in lung

Group	Cytokine (pg/mL)	1.5h Median (Min–Max)		6h Median (Min–Max)		12h Median (Min–Max)	
		Male	Female	Male	Female	Male	Female
Normal	TNF IL-1 IL-6 IL-10 TGFβ	83 (15–1031) 296 (15–734) 232 (15–617) 194 (15–376) 737 (326–887)	163 (15–806) 439 (44–7086) 95 (15–1032) 256 (15–1092) 790 (466–888)	– – – – –	– – – – –	– – – – –	– – – – –
ExSh	TNF IL-1 IL-6 IL-10 TGFβ	76 (64–185) 3299 (1147–3761)* 270 (75–438) 15 (15–210) 205 (151–510)	99 (15–134) 2671 (1540–4023) 494 (15–527) 224 (140–1088) 1291 (165–1505)	15 (15–43) 3855 (2521–5654)*** 697 (606–940) 329 (104–355) 6327 (366–6763)	15 (15–58)* 1455 (561–3180) 740 (15–1198) 52 (15–61) 3623 (3254–4709)*	ND 1835 (15–4493) 153 (15–583) 160 (15–272) 59310 (13787–62049)*	56 (23–81) 1339 (267–1529) 170 (52–483) 157 (137–692) 28180 (15847–42366)*
CLP	TNF IL-1 IL-6 IL-10 TGFβ	ND 215 (15–461) ND 45 (15–165) 282 (187–1056)	15 (15–45) 48 (15–83)* 15 (15–236) 52 (15–80) 1291 (244–2362)	89 (15–157) 145 (127–4488) 52 (15–23157) 44 (15–168) 1165 (426–3782)	62 (15–147) 214 (126–1793) 131(15–636) 196 (112–326) 2794 (1501–4502)*	15 (15–102) 68 (39–229) 15 (15–273) 47 (15–141) 16094 (11479–16588)*	15 (15–22)* 61 (15–142) ND 77 (51–152) 14017 (5000–16695)*

*TNF* Tumoral Necrosis Factor, *IL-1* Interleukin 1, *IL-6* Interleukin 6, *IL-10* Interleukin 10, *TGFβ1* Transforming Growth Factor beta

Statistical differences were considered when *p < 0.05, , ***p < 0.0005 vs Normal. N = 13 mice per N group, N = 4–5 mice per experimental group

Compared to normal mice, female mice after LPS challenge showed a significant decrease in TNF at 6 h; as seen in the liver, we observed an increasing pattern of TGFβ1 in the lungs, reaching significance at 6 h and 12 h. Male mice showed a significant increase in IL-1 at 1.5 h and 6 h; TGFβ1 also had an increasing pattern reaching significance at 12 h (Table 4).

After CLP, female mice showed a significant decrease in TNF at 12 h and IL-1 at 1.5 h compared to normal mice. Female mice also showed a constant increase in TGFβ1, reaching significance at 6 h and 12 h. Male mice also showed a constant increase in TGFβ1, reaching significance at 12 h (Table 4).

**Fig. 2:**
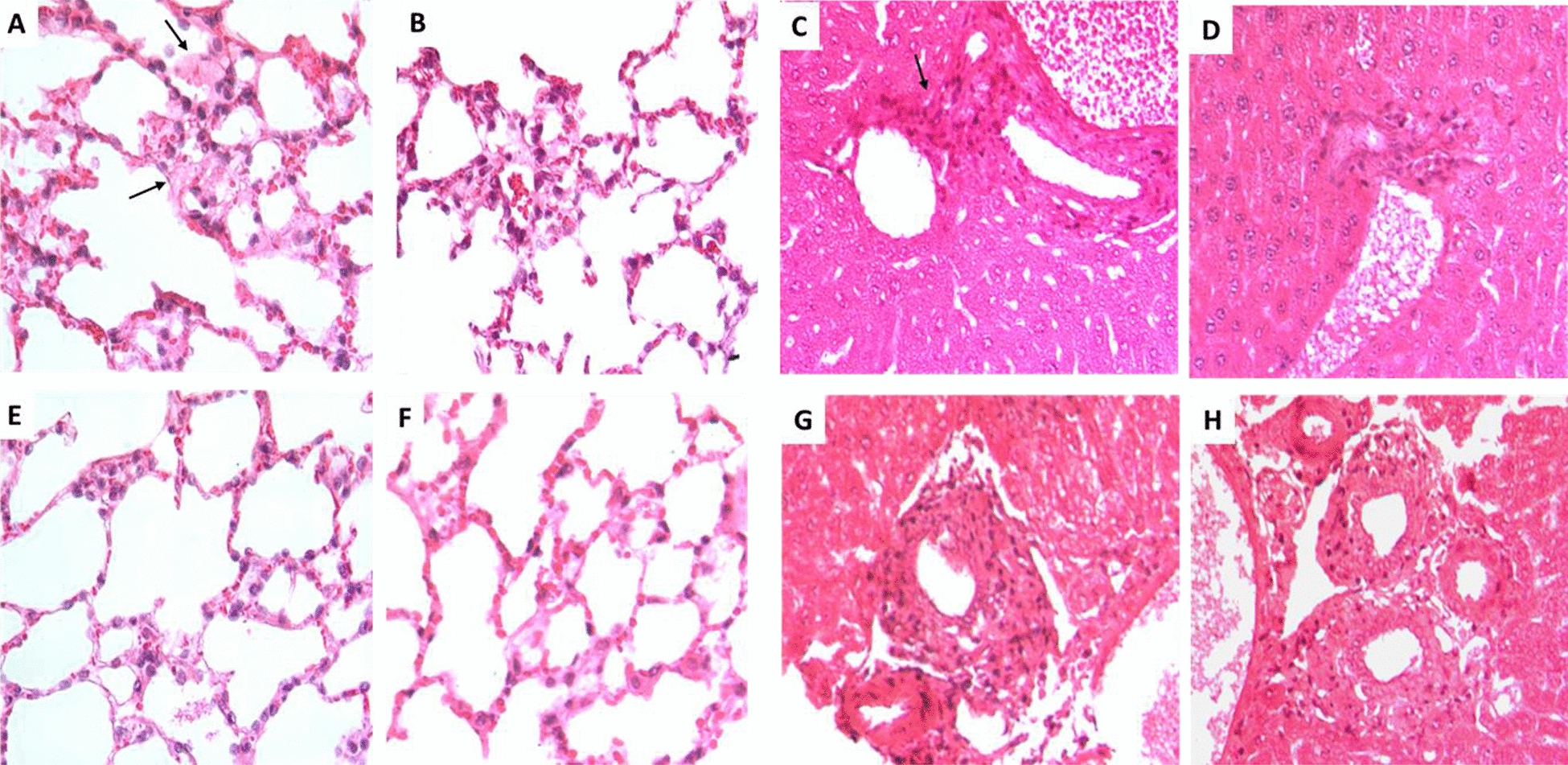
Inflammation of lung and liver from mice treated with LPS or CLP. **A** Lung section from male mice treated with LPS by the ip route, mildly sized patches of lung tissue whose alveolar lumen is occupied by acellular fibrillary acidophilic material (arrows) with few inflammatory cells. **B** Similar but fewer and smaller lesions are seen in a male mouse with CLP. **C** Liver section from male mice treated with LPS shows some portal areas with mild inflammatory infiltrate (arrow). **D** Similar lesions are seen in male mouse with CLP. **E** Compared with male mice, female mice treated with LPS show fewer and smaller areas of inflammatory infiltrate in the alveolar-capillary interstitium and scarce eosinophilic fibrillar material on the surface of the alveolar epithelium. **F** Similar lung lesions are seen in a female mouse with CLP. **G** Some portal areas show a scar-like inflammatory infiltrate in the liver of female mice treated with LPS. **H** Similar liver lesions are seen in female mouse with CLP (all sections were stained with hematoxylin and eosin, original magnification × 400). This is a representative photomicrograph of three

Histologic examination. The lungs of male mice after 12 h of LPS challenge showed occasional areas of atelectasia and mild inflammation in the alveolar-capillary interstitium with eosinophilic fibrillar material deposited on the surface of the alveolar epithelium (hyaline membranes); in some areas groups of inflammatory cells were seen embedded in this hyaline material (Fig. 2A). Female mice subjected to the same experimental procedure showed similar changes, but to a lesser extent than males (Fig. 2E). The lungs of female and male mice after 12 h of CLP treatment also showed patches of mild inflammation in the alveolar-capillary interstitial areas, with hyaline fibrillar material deposited on the surface of the alveolar epithelium, which was less extensive than in LPS-treated animals and was similar in males and females (Fig. 2B, F).

The liver of the LPS group showed a mild inflammatory infiltrate in some portal areas, which was slightly more abundant in male than in female mice (Fig. 2C, G). The liver of the CLP groups showed a similar sparse inflammatory infiltrate in some areas of the portal vein (Fig. 2D, H).

**Fig. 3:**
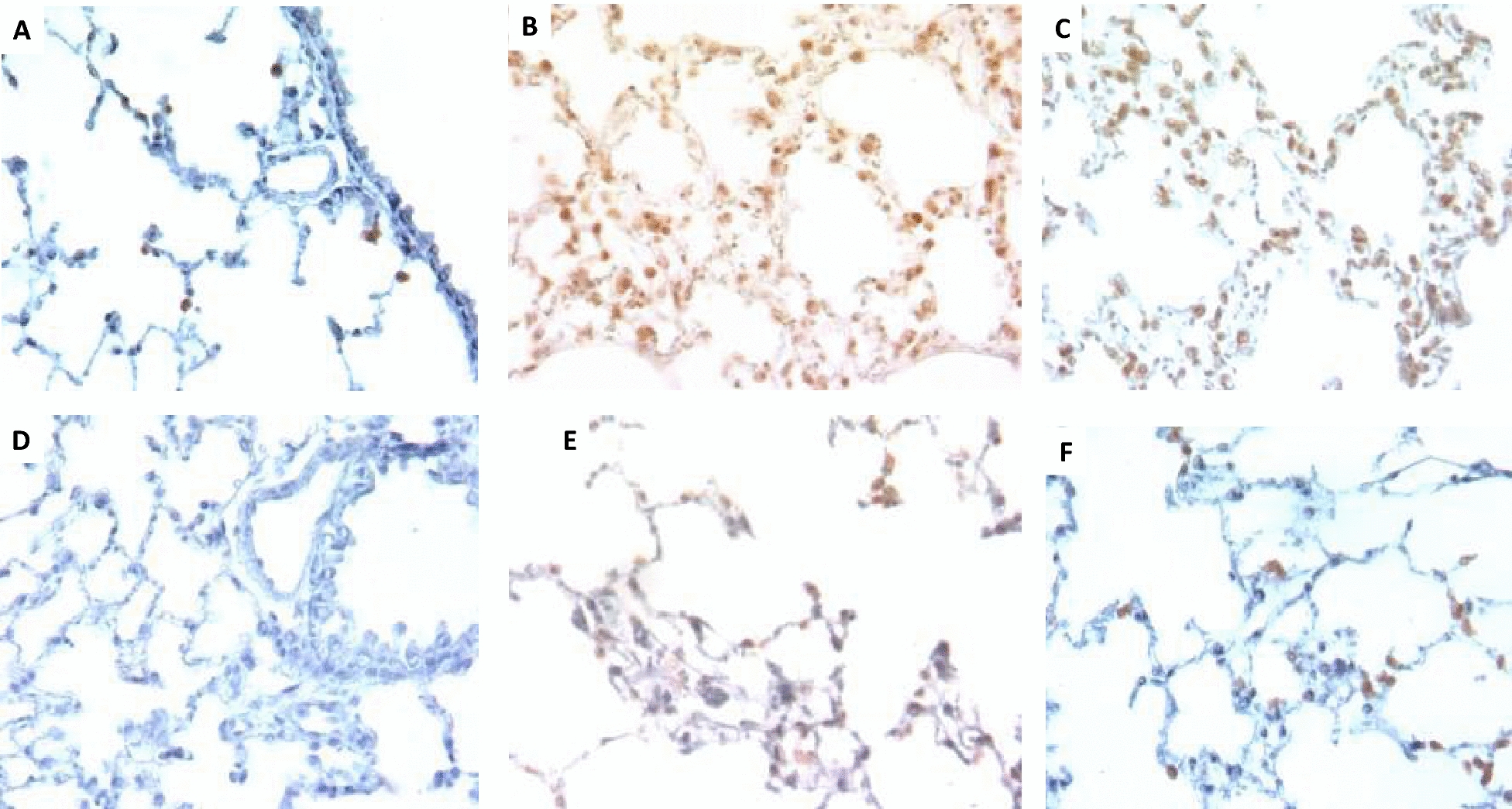
TGF detection by immunohistochemistry in the lungs of LPS- or CLP-treated mice. **A** Occasional alveolar macrophages and alveolar cells show TGF immunostaining in the lung of a control female mouse. **B** In contrast, numerous TGF-immunostained cells are present in the alveolar capillary interstitium of the lungs of female mice treated with LPS. **C** Numerous TGF-immunostained cells are also present in the lumen and alveolar capillary interstitium of the lungs of CLP-treated female mice. **D** The lungs of male mice do not show TGF-immunostained cells. **E** The alveolar space and walls of the lungs of male mice treated with LPS show some TGF-immunostained cells. **F** Similar TGF immunostaining pattern is seen in the lungs of mice after CLP procedure. (all photomicrographs × 400 magnification). This is a representative micrograph of three

While immunohistochemical detection of TGFβ1 showed similar results in the liver of all groups (data not shown), occasional positive alveolar macrophages and alveolar epithelial cells were observed in female N mice (Fig. 3A). No TGFβ1-immunostained cells were observed in male mice (Fig. 3D). Female mice treated with LPS showed numerous macrophages and lymphocytes with strong TGFβ1 immunostaining distributed in the alveolar-capillary interstitium, particularly near areas of hyaline membrane deposition (Fig. 3B); similar cellular immunostaining and distribution, but to a lesser extent, was seen in female mice treated with CLP (Fig. 3C). The lungs of male mice treated with LPS showed lower levels of TGFβ1-immunostaining cells (Fig. 3E), and even lower levels were seen in male mice with CLP (Fig. 3F).

## Discussion

Sepsis is a catastrophic syndrome that begins when bacteria or their antigens, such as LPS, emanating from an infectious site induce an exaggerated systemic inflammatory response responsible for tissue damage in target organs and often a fatal outcome. Currently, it is undeniable that females have differences in the immune response to infection and that these mechanisms provide greater resistance to infection compared to male mice. In this work, we investigated early immunologic and histopathologic changes induced by ExSh or CLP models in female and male mice under the assumption that early pathologic events influence the development and outcome of sepsis. In this study, we provide evidence of different peripheral and local inflammatory responses between models and sexes, revealing a possible protective role of TGFβ1 in females with ExSh.

As a consequence of septic challenge, the mice developed hypothermia, tachycardia, tachypnea, piloerection, lethargy, decreased blood volume and death; these signs were more evident in males and principally in the ExSh model. However, the first interesting finding was that the lethal dose of LPS for males [14] was sublethal for female mice. It was necessary to double the dose to achieve a female mortality close to 100%; these results support our previous observations where the LD100 for female mice was 250 mg/kg LPS [15]. In contrast, no sex differences in mortality were observed in CLP-induced sepsis, where puncture was performed with a 21G needle. Previous independent studies in this model have reported mortality of 20–50% in female mice [16, 17], while mortality in males was 70–90% [18, 19]; in our laboratory, mortality was 30–50% within the first 4 days [20, 21]. These differences in survival are not surprising, as they could depend on the mouse strain, age, weight, number of cecal perforations, etc. [8, 22]. Our results are consistent with those of other studies where no significant sex differences were found between 7 and 16 days after CLP [23, 24]. ExSh is more lethal than CLP in male mice. Evidence suggests that estrogen increases resistance to bacterial infections, regulating inflammation. A study in which endogenous estrogens were removed showed a marked increase in the severity of *Mycobacterium avium* infections; this result could be reversed after 17β-estradiol replacement [25]. In ovariectomized CLP-challenged rats, increased serum levels of IL-6 and TNFα are reduced after treatment with progesterone [10]. Similarly, in ovariectomized mice challenged with LPS, increased levels of IL-6 and LPS-binding protein (LBP) and a proinflammatory cellular response were shown, while estrogen treatment suppressed acute inflammation [26, 27]. Intact or ovariectomized mice treated with estradiol and challenged with intratracheal LPS, regulate acute lung injury, PMN recruitment, and IL-6 and IL-1β production in vivo and in vitro [28]. In ovariectomized mice, estrogen treatment reversed LPS exposure-induced mortality [29]. In contrast, in orchiectomized male mice, testosterone treatment increased endotoxin-induced inflammation [30]. Androgenized females have a higher mortality rate after LPS administration [29]. Finally, in vitro, treatment with 17β-estradiol to murine peritoneal macrophages (MPMs) from ovariectomized females significantly reduced the relative gene expression of TNF, IL-1 and IL-6 compared to MPMs from untreated females; similar results were obtained in MPMs treated with 17β-estradiol compared to MPMs from untreated males [30].

Another difference was seen in free circulating LPS levels, the highest levels were seen in ExSh mice, suggesting that 12 h is a short time to clear circulating endotoxin. On the other hand, low levels of endotoxin were detected in the CLP model, suggesting that live bacteria release discrete concentrations of free LPS, but this could be present for a longer period of time as the bacteria gradually and continuously enter the peritoneal cavity until cecal perforations are sealed [31]. In response, as a function of LPS levels and probably in an effort to neutralize it, natural IgM anti-LPS antibodies decreased in ExSh of both sexes compared to N groups [32]. Since this mechanism was not specific to females, it is unlikely to be responsible for the protection in the endotoxemia model; antibodies in the CLP group were discretely reduced compared to those in the N group, strengthening the idea that the polymicrobial infection releases less LPS. The presence of bacteria in the blood was demonstrated only in the CLP model, with colony forming unit (CFU) counts similar in both sexes and not associated with differences in survival. Because ExSh was a noninfectious challenge, CFUs were not detected.

The high lethality induced by endotoxemia in males was associated with an inflammatory profile characterized by high peripheral levels of TNF and IL-6; this was also consistent with inflammation in the liver and lungs, where significantly high levels of TNF at 1.5 and 12 h and of IL-6 at 6 h or of IL-1 during the first 6 h were found. In contrast, males had low levels of IL-10 in sera and tissues and delayed production of TGFβ1 in liver and lungs. This pathway, in which endotoxin is recognized by TLR4 of macrophages, monocytes, neutrophils and tissue cells such as hepatocytes, endothelial cells, etc., has been described as responsible for the pathophysiology of sepsis. The activation pathway continues with phosphorylation of inhibitor of NF-κB (IκB), its ubiquitination and subsequent degradation by proteosomes; p65:p50 dimers translocate to the cell nucleus and promote the synthesis of inflammatory molecules such as TNF, IL-1 and IL-6 [33], activating damage mechanisms such as inducible nitric oxide synthase (iNOS) and overproduction of nitric oxide (NO) or overproduction of reactive oxygen species (ROS) [34, 35]. The former is responsible for the generation of reactive nitrogen species (RNS) in sepsis, which initiate an ONOO- (peroxynitrite)-mediated mechanism and thus contribute to nitrative stress and endothelial dysfunction [36, 37], while ROS levels are mediated by the NADPH oxidase complex and mitochondrial ROS production. The targets for ROS/RNS damage include biomolecules such as proteins, lipids, and DNA, and induce cytotoxicity leading to multiple organ damage. In addition, the role of superoxide anion includes endothelial cell damage, increased microvascular permeability and amplification of the inflammatory process [35]. In another hand, tissue factor (TF) release as a consequence of endothelial injury interacts with inflammatory cytokines IL-1, IL-6 and TNF, upregulating the cascades of procoagulant pathways. Dysregulated thrombus formation can lead to coagulation factor depletion and the development of disseminated intravascular coagulation (DIC) and tissue damage, triggering pathogenic events such as stroke, deep vein thrombosis, and acute respiratory distress syndrome [38]. These mechanisms contribute to amplification of the inflammatory process, tissue injury, multi-organ failure (MOF) and death. In ExSh female mice, systemic and local inflammation was reduced, which was associated with a significant increase in TGFβ1 in the liver throughout the study (1.5–12 h) and in the lungs after 6 h.

These results suggest that TGFβ1 is an important anti-inflammatory protector in the target organs of ExSh female mice. Although three isoforms of TGFβ1 have been described in mice, TGFβ1 is the most important because it is produced in organs such as the lung, liver, spleen, and lymph nodes in mice [39]. Evidence suggests that TGFβ can regulate inflammation through several mechanisms, including (1) cell migration; mice with deletion of the TGFβ gene develop spontaneous organ failure associated with mononuclear cell infiltration [40]; (2) receptor and cytokine expression; TGFβ inhibits CD14, IL-1β and TNF expression in LPS-stimulated mouse macrophages [41] and regulates signaling pathways in myeloid cell activation by innate receptors or cytokines by inhibiting NF-kB signaling [42]; (3) polarization towards M2 type; when TGF-secreting mesenchymal stem cells (MSCs) and RAW264.7 cells are co-grown peritoneal macrophages stimulated with LPS, decreased IL-6, IL-1β and iNOS expression and production and increased IL-10 and ARG-1 compared to endotoxin-only stimulated; however, the effect of MSCs was significantly reduced by a TGFβ R inhibitor, suggesting that the reduction in inflammation was due to TGFβ secretion [43]. (4) Regulate the proliferation and differentiation of Th1, Th2 and cytotoxic T cells and promotes the development of Th17, Th9 and T follicular helper cells and (5) strongly influences the function of monocytes and macrophages, downregulating their pro-inflammatory functions [44]. The role of TGFβ1 in sepsis is controversial, but it is a regulator of inflammatory function. TGFβ1 blocks endotoxin-induced hypotension and improves survival in rat models, it decreases the number of neutrophils during the onset of LPS-induced acute lung injury and promotes the release of IL-6 from mast cells, which promotes neutrophil clearance [45]. It has been reported that TGFβ1 null mice and mice lacking the TGFβ1 transcription factor Smad3 develop spontaneous inflammatory lesions, suggesting that this mediator is fundamental in controlling innate immune mechanisms [46]. In a rat model of endotoxemia, TGF-β1 inhibited hypotension by reducing iNOS expression in the heart, kidney, and liver [47]. Furthermore, TGFβ1 production is reduced in αβ T-cell deficient mice, leading to increased production of TNF and IFNγ [48]. On the other hand, histopathological damage in liver and lung was ameliorated by injection of mesenchymal stem cells with high TGFβ1 expression 6 h after induction of sepsis by CLP [49]. In our study, early expression and production of TGFβ1 in ExSh female mice could regulate inflammation and attenuate target organ injury. Nevertheless, treatment of mice with anti-TGFβ antibody attenuated leukocyte migration, vascular permeability, and increased survival, as reported by Bae et al. [50]. These findings suggest that this cytokine may have a dual role in sepsis, early as an immunoregulator or late as a promoter of inflammatory mechanisms. Undoubtedly, the timing and dose of anti-inflammatory versus pro-inflammatory molecules is fundamental to attenuate or enhance the inflammatory response. In ExSh, the challenge is acute and lethal because mortality is rapid. Although we do not know the exact mechanism of the early production of TGFβ1 in females after ExSh, it could play an immunoregulatory role that attenuates the injury in target organs. In CLP, the challenge is chronic and damage and lethality are slower, which may be why we didn’t find differences in mortality between the sexes and the immunoregulatory effect of TGFβ1 was not clear. Few studies have examined the levels of TGFβ1 in both sexes, in the serum of normal healthy blood donors, it has been reported that the levels do not correlate between the sexes at different ages [51]. However evidences suggest a close relationship, several studies support that female sex hormones favor TGFβ production, as evidenced by a clear increase in TGFβ1 compared to controls when epithelial cells are cultured in the presence of progesterone [52]. Although, estrogens have been proposed to prevent inflammatory gene transcription by inhibiting intracellular transport of NF-κB as an immediate-early mechanism in the inflammatory signaling cascade [53]. In vivo, local application of 17β-estradiol to aged human skin results in increased TGFβ mRNA expression in aged female skin compared to aged male skin; this difference is maintained upon immunohistochemical testing [54]. In ovariectomized rats, low TGFβ production by bone cells is observed, whereas replacement therapy with 17β-estradiol directly stimulates TGFβ production [55]. Finally, in vivo, similar results have been shown in ovariectomized rats, which showed a significant decrease in TGFβ levels compared to their control group, while estrogen-treated rats had a higher blood TGFβ concentration compared to the ovariectomized group, suggesting that estrogen deficiency suppresses TGFβ expression and, in this study, interfered with fracture healing [56].

We believe that future studies need to evaluate the role of TGFβ1 and female sex hormone as a possible combined protective mechanism in sepsis.

On the other hand, low lethality shown in male CLP mice, was associated with a discrete and late inflammatory profile in serum and liver, surprisingly, TGFβ1 levels increased in liver of male mice at 6 h and in lung at 12 h, while in serum of female mice cytokine levels had in N group levels, while TGFβ1 levels increased at 12 h in liver and starting from 6 h in lung of female mice. Discrete inflammation in CLP mice, support that the injury is chronic as a result of less LPS free in CLP model. Interesently, low concentrations of TNF, IL-1 and IL-6 in target organs homogenized in both models of sepsis compared to the levels measured in N mice, we propose that four mechanisms were involved for these results, the first is the contribution of tissues for high levels of cytokines in the blood; second, the uptake of cytokines by target cells or tissues [57–60]; thirdly, the flux of inflammatory mediators into the interstitial space as a result of the loss of endothelial barrier function and the increase in tissue permeability [5, 61, 62]; finally, molecules such as sTNFR, IL-1Ra and sIL-6R, which could compete for their circulating free ligands and their detection by immunoassays, should not be excluded [63–65].

## Conclusions

In this study we were able to show clear differences between the models. First, there was a sexual dimorphism in ExSh, where males were more susceptible than females from 24 h onward. Females resisted twice the LD100 used in males. However, no sex differences in mortality were observed over four days in the CLP model. Second, antibody neutralization was not sufficient for protection because LPS administration induced high circulating levels of endotoxin and a consequent rapid depletion of natural anti-LPS antibodies. In the CLP model, the levels of antibodies and endotoxin were low. Third, high levels of LPS in male mice were associated with early inflammation and injury in target organs. Finally, early lung TGFβ1 production in ExSh, but not CLP, females was associated with increased survival and decreased lung injury.

Limitations. We are aware of the limitations of the use of animal models, however ExSh and CLP continue to be used in preclinical studies because both reproduce different immunopathological mechanisms developed in human sepsis; however, there are no comparable conditions in both models to induce equal mortality. In ExSh there is a clear greater susceptibility for males than females, but in CLP there were no differences and puncture was not tested with needles larger than 21G. We measured classical mediators; however, we do not know whether chemokines, sex hormones, or other cytokines may play an important role in the development of early injury in the different models and sexes. We also did not measure soluble or antagonistic receptors that may be involved in regulating the inflammatory response in female mice with ExSh.

## Methods

### Endotoxic shock model (ExSh)

Female and male Balb/c mice aged 8–12 weeks old and weighing between 21–23 g and 25–27 g, respectively, were housed under standard laboratory conditions with food and water ad libitum and light/dark cycles (12/12 h). These animals were challenged intraperitoneally with a lethal dose 100 (LD100) for males (25 mg/kg) of LPS serotype 055:B5 (Sigma-Aldrich) or 50 mg/kg for females (close to LD100) [14].

### CLP model

Female and male Balb/c mice aged 8–12 weeks old and weighing between 21–23 g and 25–27 g, respectively, were housed under standard Biotherium conditions with food and water ad libitum and light/dark cycles (12/12 h). For the surgical protocol, mice of both sexes were anesthetized intraperitoneally (IP) with pentobarbital sodium (25 mg/kg). A 1–2 cm midline incision was made, the cecum was exposed, its base was ligated with 4–0 black silk sutures without occlusion, and then punctured twice with a 21-gauge needle. Finally, the cecum was crushed and returned to the peritoneal cavity. The wound was sutured in two layers (muscular and dermal) with 4–0 black silk. Our protocols were reviewed and approved by the local ethics committee with registration number 11-726 [20, 21].

### Survival

A batch of animals, including males and females with ExSh or CLP, was used to assess survival, daily for 1 week.

**Sample collection**. Another batch of animals was used to collect blood and organs; these animals were previously anesthetized intraperitoneally (IP) with an overdose of sodium pentobarbital (50 mg/kg). Blood was collected by cardiac puncture in different mice 1.5, 6 and 12 h after endotoxic shock (ExSh) or CLP, processed in cold and centrifuged at 2500 rpm for 15 min at 4 °C. Serum was separated and stored at -75 °C. Livers and lungs collected at the same time points were harvested and frozen at − 75 °C.

### Tissue homogenization

100 mg of livers and lungs were homogenized in cold in 1 mL PBS with a cocktail of protease inhibitors (aprotanin 1 µg/mL, leupeptin 1 µg/mL, pepstatin A 1 µg/mL, and PMSF 100 µg/mL) using a Polytron, samples were centrifuged at 2000 rpm at 4 °C, and supernatants were collected. Proteins were quantified from the supernatants using a Pierce BCA protein assay kit (Thermo Scientific, CA, USA) according to the manufacturer's instructions. Cytokine levels were normalized per mg of homogenized tissue.

### Measurement of cytokines

TNF, IL-1β, IL-6, IL-10, and TGFβ1 levels in serum and liver and lung supernatants were measured by ELISA (R&D Systems, Minneapolis, MN, USA) according to the manufacturer's protocols. Tissue normalization was expressed as pg cytokines/mg total protein. When cytokine levels in samples exceeded the upper limits of the assays.

### Bacterial quantification

To assess the presence of bacteria in the circulation, blood was obtained by cardiac puncture from another group of animals previously anesthetized with sodium pentobarbital, serially diluted tenfold in sterile saline, and cultured overnight in 5% sheep blood agar (Teknova, Hollister, CA, USA). Colony-forming units (CFU) were quantified by manual counting.

### Detection of IgM anti-LPS antibodies

IgM anti-LPS antibodies (Abs) were detected using an ELISA modified from Pollack et al. [66]. Briefly, Maxisorb plates (NUNC) were sensitized overnight at 4 °C with 50 μL antigen solution (LPS diluted to 25 μg/mL in 15 mM Na_2_CO_3_, 3 mM NaN_3_, pH 9.5 coating buffer). Sera collected 12 h after CLP were diluted 1:64 in the buffer provided in the Mouse Typer Sub-Isotype Kit (Bio-Rad), and the IgM isotype was determined using rabbit anti-mouse antibodies according to the manufacturer's instructions.

**Histological examination**. After 12 h of ExSh or CLP, some mice of both sexes were sacrificed and their livers and lungs were fixed by immersion and perfusion in absolute alcohol, respectively, and embedded in paraffin. 10 μm sections were stained with hematoxylin and eosin. The proteinaceous eosinophilic material deposited on the surface of the alveolar wall (hyaline membranes, indicating pulmonary edema with diffuse alveolar damage at 200X magnification) was evaluated. In liver sections, the inflammatory infiltrate was evaluated at 200 × magnification. The presence of cellular infiltrates was evaluated in both tissues.

Local production of TGFβ1 was determined by immunohistochemistry using the same paraffin-embedded lungs. Tissue sections of 4 μm width were cut and mounted on poly-l-lysine-coated slides (Biocare Medical). Tissue was hydrated by passing the slides 5 times through the following solutions: xylol, xylol-alcohol (1:1), absolute alcohol, 96% alcohol, and distilled water. Heat-induced antigen retrieval was performed using ImmunoDNA Retrieve 1 × with Citrate (Bio SB). Endogenous peroxidase was blocked with 3% hydrogen peroxide. Washes were performed with PBS-Tween 20 0.05%. The tissue area was outlined and blocked with 200 μL Background Sniper (Biocare Medical) and incubated in a humidity chamber for 20 min. Tissue sections were incubated with goat polyclonal antibody anti-TGFβ, dilution 1:100 (sc-402, Santa Cruz Biotechnology) overnight at room temperature with shaking. After PBS washing, sections were incubated with goat anti-rodent HRP polymer (GHP516L, Biocare Medical) as secondary antibody for 30 min. In both cases, antibody binding was detected with 200 μL diaminobenzidine (ImmPACT DAB Substrate Kit, Peroxidase) and counterstained with hematoxylin [67].

**Statistical analysis**. All results are expressed as medians (Min–Max). Comparisons of survival curves were performed using the Mantel-Haenzel log-rank test. For comparisons with ND results, the minimum sensitivity of the ELISA assay was used, and comparisons between groups were performed by Kruskal–Wallis test (post hoc analysis by Dunn's test) and Matt-Whitney test. A value of p < 0.05 indicates significance. GraphPad Prism 8.0 statistical software (GraphPad Inc, San Diego, CA, USA) was used. The authors declare that they have no conflict of interest.

## Data Availability

Not applicable.
